# Negative chemical data boosts language models in reaction outcome prediction

**DOI:** 10.1126/sciadv.adt5578

**Published:** 2025-06-13

**Authors:** Alessandra Toniato, Alain C. Vaucher, Teodoro Laino, Mara Graziani

**Affiliations:** ^1^ IBM Research Europe, Zurich, Switzerland.; ^2^ NCCR Catalysis, Zurich, Switzerland.

## Abstract

Trial-and-error approaches in chemistry generate abundant unsuccessful experiments, yet the potential of these so-called negative results remains largely underutilized. Here, we demonstrate that information from negative chemical reactions can be leveraged to improve reactivity-prediction models, offering advantages in scenarios with a limited volume of successful data. We extend the tuning of language models with reinforcement learning to the chemistry domain, training a transformer model for chemical reaction prediction. Our approach is evaluated using both a rigorously controlled dataset and a realistic high-throughput dataset comprising extensive reaction screenings across diverse catalysts sets and experimental conditions. The model achieves state-of-the-art performance by leveraging information from as few as 20 positive data points in the controlled dataset, supported by a negative dataset at least 40 times larger. Consistent results on both datasets demonstrate that, with an appropriate optimization strategy and the inclusion of unsuccessful experimental data, models can be effectively trained even when successful reactions are underrepresented.

## Teaser

Reinforcement learning tuning of language models finally exploits negative chemical reaction data for outcome prediction.

## INTRODUCTION

Scientific advancements often emerge from a sequence of failures and subsequent learning. Thomas Edison famously remarked, “I didn’t fail 1000 times. The light bulb was an invention with 1000 steps,” acknowledging the value of failures. In the chemical science, the significance of negative data and unsuccessful reactions cannot be understated (*1*) as it provides critical insights into the boundaries and limitations of reaction conditions, which are often overlooked in traditional reporting. The utility of such data is evident in pioneering studies, where failed experiments have been used to enhance machine learning models for materials discovery, outperforming human intuition in predicting successful reaction outcomes (*2*) and in optimizing reaction conditions for complex organic reactions through closed-loop workflows (*3*). Despite the significance of negative data being widely recognized (*1*, *4*), the integration of such data into machine learning models remains challenging.

From a modeling perspective, negative experimental data are crucial for refining our understanding of models trained on positive data, particularly in regions with a low density of positive experimental outcomes. They can enhance the description of areas where models operate in an extrapolation regime due to the sparse distribution of positive data. In essence, negative experimental data reveal opportunities for improving both models and theories. The quality of unsuccessful experiments is especially important; if we consider a theory as defining a hyperdimensional manifold passing through all successful experiments, the most informative failures are those that deepen our understanding of this manifold in regions where the density of successful experiments undergoes notable changes.

**Fig. 1.**
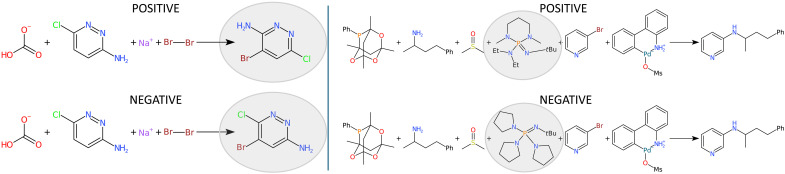
Characterization of negative data. Left: RegioSQM20 (*38*). Right: Data from (*5*). The molecular differences between the positive and negative are highlighted in gray.

Negative reactions are categorized in two types: (1) reactions yielding unexpected but chemically meaningful products and (2) reactions in which the intended product is not observed, leaving starting materials largely unreacted and indicating an unfavorable reaction pathway (*1*). Figure 1 illustrates these two categories: The left panel shows an example of a reaction that produces an unexpected yet chemically relevant product (type 1), whereas the right panel provides an example of a reaction where the anticipated product is absent, or the starting materials remain unreacted (type 2). We emphasize that type 1 negative reactions are especially valuable for refining theoretical predictions during the training of chemical language models. These cases help delineate the boundaries of model predictions by providing informative deviations from anticipated reaction outcomes. In contrast, simply generating negative reactions by pairing reactants with random, chemically unrelated products offers limited insight and does not effectively enhance model learning or clarify predictive boundaries. Type 2 negative reactions present unique challenges too. Often, it remains unclear in the literature whether the reactants and reagents failed to react entirely or simply did not yield the expected outcome, complicating their classification within the original framework (*1*). For instance, the dataset from (*5*), shown in Fig. 1 (right), represents such cases. In this work, we aim to explore both types of negative reaction data. Integrating negative data is challenging; however, we demonstrate that it can be leveraged through a reinforcement learning (RL) algorithm, leading to enhanced predictive accuracy.

The challenge lies not only in the utilization of negative data but also in the scarcity of well-characterized large-scale negative datasets. Now, most machine learning models in chemistry are trained on data from successful patented reactions (*6*), which are biased toward the positive samples (*7*). Moreover, when datasets of negative reactions do exist, they are often inaccessible or not in machine-readable format (*8*, *9*). The scarcity of negative data hampers the development of robust models capable of adapting to unsuccessful outcomes, despite the community’s recognition of their significance.

The inclusion of negative data in machine learning models for chemistry has been discussed and motivated in various contexts, particularly in reactivity prediction and reaction condition optimization. Previous studies have incorporated low-yielding reactions for reaction screening application (*2*, *9*–*13*). In addition, Bayesian optimization (BO) has been used to leverage negative data in optimizing reaction conditions (*14*–*16*). However, the application of these methods to broader reaction prediction tasks remains challenging. On the other hand, the use of negative data is well established in fields such as computer vision and natural language processing (NLP). Techniques like contrastive learning and complementary learning have been used to improve model performance either by penalizing the proximity of positive and negative examples in the embedding space, as seen in image classification (*17*, *18*) and neural machine translation (*19*–*21*) or by introducing a sign-inverted loss for negative data in multiclass classification (*22*–*24*) and language models (*25*, *26*) tasks. One widely adopted approach for enhancing model performance using negative information is through generative adversarial networks (GANs) (*27*–*30*). GANs consist of two models working antagonistically: a generator that creates realistic synthetic samples and a discriminator that evaluates these samples, classifying them as negatives for not being part of the original, human-generated data. This approach, however, has been deemed unsuitable for forward reaction prediction, where the correct product is a unique sample with a unique canonical representation, and therefore cannot be generated realistically in multiple ways. Moreover, all these approaches rely on large-scale balanced datasets, making them less effective in domains like chemistry, where data volume is an intrinsic limit.

RL inherently involves learning from failures, where models optimize their performance through trial-and-error interactions with the environment. This paradigm has been successfully applied in various language model applications, including text generation and chatbot development (*31*–*33*). Significant advancements in leveraging negative feedback have been achieved through the development of reinforcement learning from human feedback (RLHF) (*34*, *35*), in which a reward model is trained using human feedback on preferred optimization results. Subsequently, a base model is fine-tuned (FT) (*35*–*37*) to maximize the expected reward of its predictions.

In this work, we extend the concept of RLHF to the field of chemistry, demonstrating the significant value of failed experiments in training chemical reaction prediction models. By designing a reward function that accounts for both successful and unsuccessful reaction outcomes, we adjust model weights to incorporate information from negative data. We focus on two types of datasets: a highly controlled one derived from RegioSQM predictions (*38*) and a realistic high-throughput experimentation (HTE) dataset based on actual experimental results (*5*). Our findings show that, even in data-scarce environments where positive examples are limited, the inclusion of negative reactions through RL markedly enhances model performance. As a result, models trained with RL feedback not only outperform their FT counterparts but also produce a higher proportion of valid positive reactions.

## RESULTS

We evaluate the impact of integrating negative reaction outcomes into model training across two distinct data regimes: a low-data setting (RegioSQM) (*38*) and a real experimental dataset (HiTEA) (*5*). The RegioSQM dataset represents a highly controlled scenario where the available positive reactions are well characterized, and negative samples could be artificially generated. In contrast, the HiTEA dataset originates from real-world HTE and is comprehensive of reaction with various observed yields including some negative ones. Applying our approach to this additional dataset highlights the broader applicability of integrating negative data.

### RL feedback from negative data improves positive reaction prediction over FT in low data regimes

To investigate the potential benefits of incorporating negative experiments into the training of forward reaction prediction models, we compared the FT performance against our RL approach. Both methods were applied to boost the performances of a base language model pretrained for forward prediction on reactions extracted from US patent data. Specifically, RL was used to update the language model parameters of the forward model. For training, we used the publicly available RegioSQM20 dataset (*38*), which is exhaustive within a specific domain of organic chemistry, capturing all positive and informative negative outcomes. Although the dataset is confined to a narrow chemical domain, it is exhaustively characterized, making the upper bound of possible positive discoveries well known and easy to validate. This facilitates the experimentation with diverse methodologies, including exhaustive fine-tuning on all positive examples and RL strategies. From this starting point, to recreate the practical scenario where successful reactions are rare, we constructed a variation of the training set denoted as *K_low_* containing 10 times fewer positive samples than the original dataset *K_high_*, with 22 positive reactions in *K_low_* compared to 220 in *K_high_*. Both datasets include all available negative reactions.

**Fig. 2.**
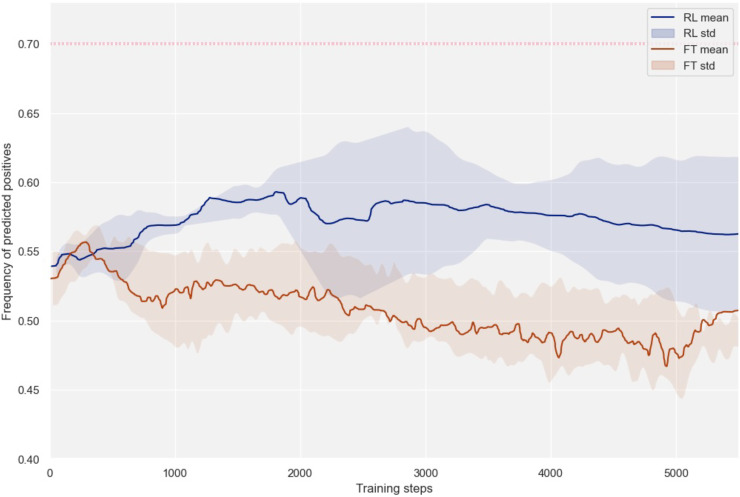
RL and FT performances in low data regimes. Frequency of correctly predicted positive reactions by the RL and the FT models trained on hundreds of failed reactions and 20 positive reactions from the RegioSQM20 dataset (i.e., the *K_low_* dataset subset). The shaded regions represent the SD across three random splits. The magenta dotted line represents the highest performance reached by FT on the *K_high_* dataset, where all the positive reactions from RegioSQM20 were used during training.

Figure 2 illustrates the positive accuracy—defined as the accuracy measured on positive samples—of models trained using the two strategies, with shaded regions indicating the SD. When all the 220 positive reactions are included (i.e., *K_high_* scenario, illustrated in the magenta dotted line in Fig. 2), FT demonstrates superior effectiveness, which is expected given the abundance of successful experiments. However, when applied to the *K_low_* dataset with only 22 positive instances, the FT approach fails to yield any improvement.

In contrast, our RL approach can successfully learn from the *K_low_* dataset, despite the limited number of positive samples, ultimately surpassing the performance of FT. The strength of the RL approach lies in its ability to shift the challenges associated with the scarcity of positive data and the abundance of negative samples from the language model to the reward model. Central to our RL implementation is the strategic selection of a reward model that can be effectively trained with a minimal number of positive instances. This approach enables the framework to excel in identifying high-quality positive predictions as they emerge during RL, which are then used to guide the training of the language model through a gradient-based policy.

Table 1 presents the accuracy comparison between the RL and the FT models over five repetitions on different cross-validation splits, on the *K_high_* and *K_low_* datasets as described in Table 2. The results corroborate the observations from Fig. 2, showing that the RL approach is particularly effective in low data regimes. In addition, results on the test set of the US patent dataset (i.e., USPTO) indicate that the RL models at worst maintain the original performance, avoiding the phenomenon of catastrophic forgetting (*39*).

**Table 1.** Positive accuracy () of FT and RL models on RegioSQM20 and the USPTO test set. ↑ The SD is reported in parentheses. Only the best accuracy on *K_low_* is highlighted. Comparisons are column-wise. The asterisk (*) on FT-*K_low_* indicates no boost in performance observed because the accuracy coincides with that of the starting forward model.

% Positives	FT models	USPTO	RegioSQM20	RL models	USPTO	RegioSQM20
100%	FT-*K_high_*	57.98 (±0.13)	68.48 (±1.38)	RL-*K_high_*	58.87 (±0.05)	63.15 (±1.64)
10%	FT-*K_low_*	59.43 (*)	54.91 (±1.04)	RL-*K_low_*	59.22 (±0.08)	58.55 (±1.75)

**Table 2.** Data breakdown. Number of positive (pos.) and negative (neg.) reactions and their ratio in *K_high_* and *K_low_*.

Data	Train set			Valid. set	Test set
	Pos.	Neg.	Ratio	Pos.	Pos.
*K_high_*	220	748	0.3	165	164
*K_low_*	22	748	0.03	165	164

### Highly generalizable reward functions enhance RL feedback quality

The reward model is a crucial component of our method because it allows us to identify which reactions among the predicted ones are likely to be positive. This benefit is not directly evident on the *K_high_* dataset due to the overabundance of positives in the training data facilitating the learning through FT. However, when the positives are scarce (such as in *K_low_*), RL is better at discovering positives because the reward model is bringing additional information on what reactions could potentially be unobserved positives. To achieve this result, we built a reward model that classifies positive from negatives chemical reactions while accounting for the strong underrepresentation of the positives in the training data.

The representation of positive and negative data plays a pivotal role in this process, as the choice of representation space can significantly influence the separation between these two classes, thereby enhancing the effectiveness of the reward function. In our approach, we used embeddings that form the basis of reaction fingerprints (*40*). We explored two distinct strategies: The first involved using the embeddings from the base language model used for the reward calculation; the second used the embeddings obtained by fine-tuning the same base language model on the downstream task of reaction classification into successful and unsuccessful reactions. The dataset used for this fine-tuning included a small subset of positive and negative USPTO sequences that were not seen during the pretraining of the base model nor used during testing. Both representations were then used to train a support vector machine (SVM) to identify positive predictions generated by the RL model. This strategy smartly exploits the generalization potential of simple models, enabling the RL to learn how to transform incorrect negative predictions into other positive ones. Step-by-step details about model training are discussed in the Materials and Methods and in the Supplementary Materials.

**Fig. 3.**
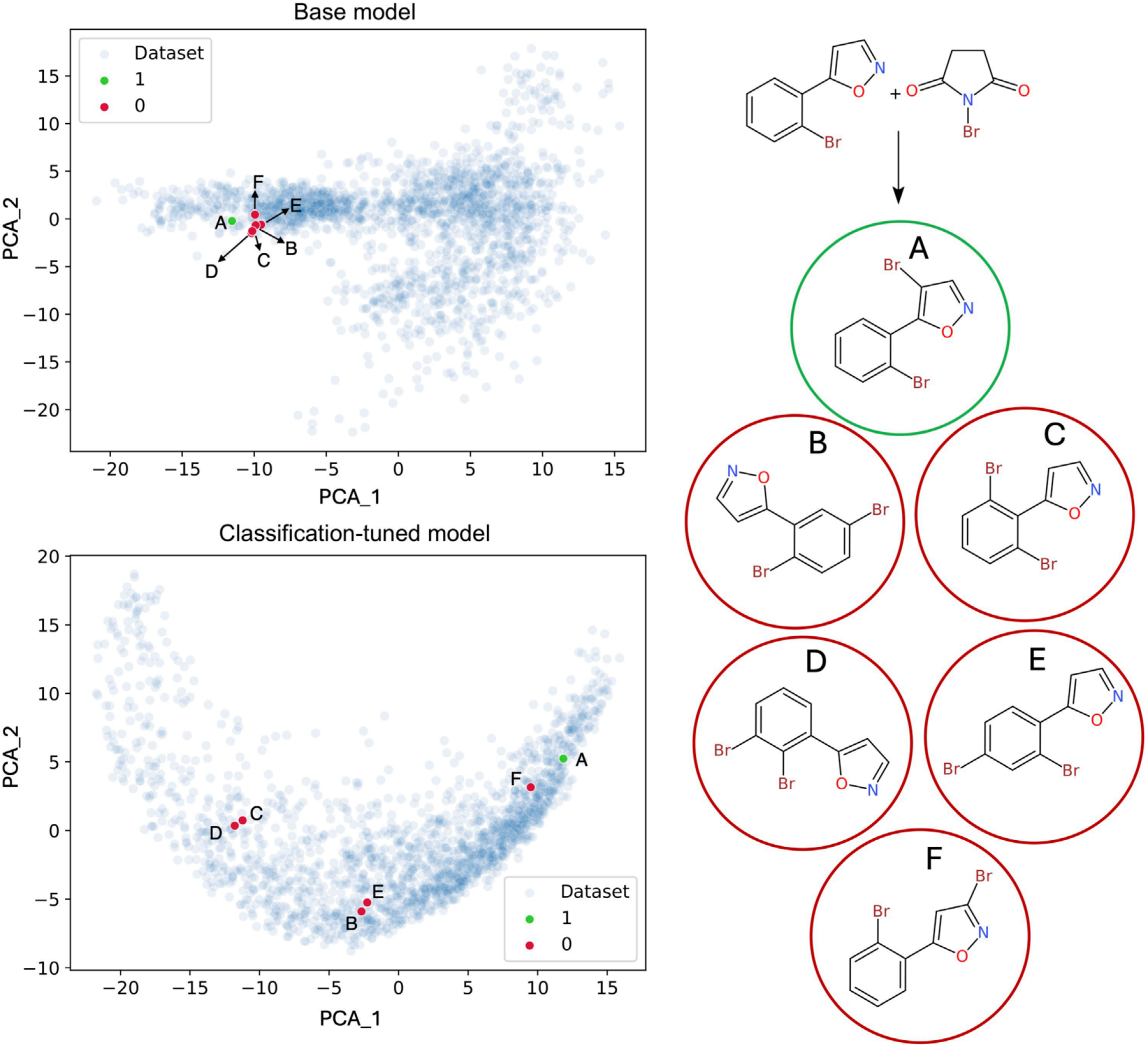
Illustration of positive and negative embedding vectors for the bromination reaction of 5-(2-bromophenyl)isoxazole with *N*-bromosuccinimide. In the case of the base model (top left), negative reaction outcomes are tightly clustered to positive reaction outcomes, whereas for the classification-tuned model (bottom left), negatives are cast further apart from the positive. Blue points are the rest of both RegioSQM and USPTO. A is the correct product of the bromination reaction. B, C, D, E, and F are negative products.

Figure 3 illustrates the impact of training the base reward model to classify positives and negatives derived from the USPTO. The visualizations on the left represent the first two principal components in the two representation spaces, namely, the one of the base model (top) and the one obtained from classification tuning (bottom). In the latter (bottom), positives and negatives appear more separable than in the base model embeddings. Such separation, which is quantified in table S2 of the Supplementary Materials, proved essential to the training of the SVM for the reward model.

### Impact of data variability

Training a model using RL remains challenging for the scientific community, primarily due to the complexity and stability issues associated with the process. When training a RL model, multiple models contribute to the learning process, with the policy and the reward model adding complexity to the process of tuning the forward model. Such complexity introduces additional sources of variability, for which it is important to test the model robustness and the consistency of the results under different conditions. In a previous work, we tested the forward model on different initializations while keeping the data fixed, noting no significant variability in the observed performance (*41*). For this reason, we focused on assessing the stability of the results when variability is observed in the training and validation datasets used to tune the models.

**Fig. 4.**
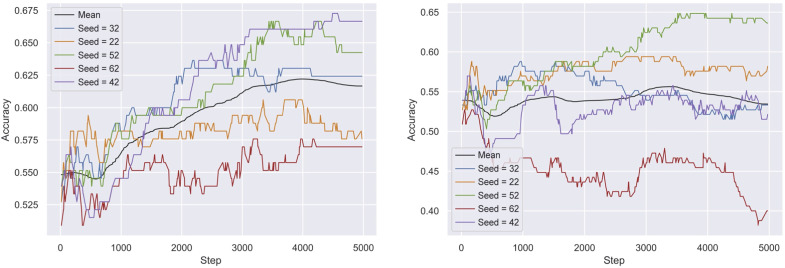
Breakdown of RL performance. Positive accuracy of RL trained on data splits obtained from five starting seeds for *K_high_* (left) and *K_low_* (right).

Variation in performance is observed in both the FT and the RL models (see fig. S2 in the Supplementary Materials). Particularly in the *K_low_* dataset, an unfavorable data split may hinder the SVM’s ability to identify positives, potentially degrading the learning process. However, the breakdown in Fig. 4 illustrates that this happens for one of the five tested data splits, specifically the one associated with seed 62. This split consistently yielded the lowest performance across both our proposed RL approach and the FT baseline. The reward SVM model showed the poorest overall performance on this data split (see table S1 of the Supplementary Materials), with a training accuracy of 69.57% and a test accuracy of 64.24%. Because the performance challenges were consistent across models, we attribute the instability to intrinsic data variability. Besides, the FT baseline showed low performance in an additional data split (seed 22), which was better handled by our RL approach (see fig. S2 in the Supplementary Materials).

The model interdependencies are one of the challenges of implementing RL, where factors such as hyperparameter selection or data splitting can critically affect outcomes at different stages of the development process (*42*). For this reason, it is critical to approach the implementation of the RL approach with careful consideration and attention to detail.

### Applicability to HTE data

HTE data consist of real-world experimental results that explore the space of chemically and physically feasible reactions, with the purpose of detecting under which conditions a certain target compound is formed. These data are typically collected by systematically varying reactants and reagents across a predefined set of possibilities, assigning them to the categorization of negative data of type 2, as discussed in Introduction. For example, an array of catalysts and solvents can be screened with the same reactants to analyze the yield of specific compounds in the final reaction mixture (*4*). To test the applicability of our method to this distinct task, we expanded our experiments to the HiTEA dataset (*4*). Specifically, this comprehensive collection of reaction conditions allowed us to explore how our method performs on this type of experimental data, which presents unique challenges compared to those observed in RegioSQM.

First, because of the different purpose of HTE, the HiTEA dataset includes a significantly lower number of explicitly competing reactions where the same left-hand side led to distinct products of which we could label one as negative. Only a small fraction of the high-yield products contained a negative yield counterpart originating from identical reactants (i.e., 9 of 130). In addition, the statistical coverage of the dataset is not uniform, with some reactants and reagents being over- or underrepresented in the dataset (*4*).

To address these challenges, our RL approach was adapted to encourage the prediction of reactions for which the observed yield was higher than 1%, simultaneously discouraging the predictions of reactions that we labeled as negative because of low observed yield. We emphasize that the choice of a 1% threshold was chosen solely to assess the model’s ability to learn from this dataset categorization. Although such a threshold may have important implications in the domain of real chemical experimentation, its relevance here is limited to demonstrating the applicability of our approach to leverage negative data. The results from our RL approach suggest that the prediction of high-yield reactions can be boosted by RL tuning. After fine-tuning the forward model on this refined collection of reactions to address the strong initial domain shift from the USPTO, we tuned the model weights with our RL pipeline. Particularly in the case of *K_low_*, where we focused on reactions that are underrepresented in the original dataset, the RL model boosted the FT model accuracy, reaching a positive accuracy on the validation set of 0.644 (±0.015) against the FT validation performance of 0.610 (±0.018). The SD, reported in parentheses, reflects variability across different training data splits and for varying initial hyperparameter configurations. The maximum accuracy reached by the RL pipeline was 0.668 against 0.628 of the FT. The boost given by the RL is observed consistently also in the test set performance (top 10 predictions), which is reportedly at 0.441 for the RL against 0.427 for the FT model. When the training is extended to *K_high_*, hence to the full range of available positive examples, the same performance boost is observed, with the RL model reaching an average positive accuracy of 0.891 (±0.001) against the FT model reaching 0.877 (±0.001).

A further analysis of the model predictions reveals that such boost is obtained by the action of the reward model, which discourages the predictions of low-yielding reactions (i.e., yield < 1%) and encourages the prediction of high-yield reactions. In-depth descriptions of the modeling steps and techniques, convergence plots, and additional results are given in the Supplementary Materials (figs. S5 to S7).

## DISCUSSION

The integration of negative chemical data in the training of machine learning models has been a long-standing challenge in the community. This work shows that language models for predicting chemical reactions can be improved by learning from negative (unsuccessful) reactions. It uses insights gained from these negative examples to better characterize the learned manifold of successful (positive) chemical reactions. We demonstrate the potential of incorporating feedback from negative chemical data in the training of language models through RL and demonstrate that this is a valid alternative as opposed to model fine-tuning, both in low data regimes and in real HTE datasets. The first objective of this work was to investigate reactions with clearly defined negative outcomes. These well-controlled examples offer a unique opportunity to expose the model to fundamental failures, refining its understanding of chemical rules. The second objective focused on demonstrating the practical applicability of our approach using the HiTEA dataset. In this context, we used a binary reward function to distinguish between successful and failed reactions. Although effective, this setup also opens the possibility for future extensions, such as designing custom reward functions that factor in experimental details like reaction yield—ultimately leading to more robust and realistic predictive models. To achieve this, one would need in-depth analyses on how to appropriately account for reactions with low but nonzero yields.

Moreover, we observed some inherent instability of the RL training, which requires a meticulous approach to hyperparameter selection. Additional robust strategies are required to determine the optimal hyperparameters ranges, which should be tuned on average performance across multiple seeds, rather than relying on a single, randomly chosen seed (i.e., seed 42). In addition, increasing the number of seeds used to split the dataset is crucial, although it substantially increases the computational demands of model training.

These findings not only deepen our understanding of RL model development in chemistry but also pave the way for broader applications across various chemical tasks and scientific disciplines. In regions of the feature space with scarce positive data and abundant negative data, RL strategies allow models to more accurately capture the underlying physical laws governing well-represented regions while also improving the description of those areas with lower positive data density. The inclusion of meaningful negative data refines these regions, complementing positive data and providing a more comprehensive understanding of the governing principles of a given problem.

## MATERIALS AND METHODS

### Experimental design

The datasets and models used for the experiments are outlined below.

### USPTO dataset

The baseline dataset is the original USPTO dataset, comprising reactions extracted from US patents (*6*). We performed basic cleaning and standardization procedures on the SMILEs strings (*43*), including canonicalization (*44*), removal of duplicates and residual reactants on the product side, and exclusion of invalid reactions. In addition, we removed from the dataset any products that matched those present in the FT dataset described below, ensuring no overlap of positive reactions with the baseline dataset. Following an ~10% split, the dataset consisted of 396,145 reactions for training and 49,517 and 49,518 reactions for validation and testing, respectively.

### RegioSQM20 dataset

The FT datasets *K_high_* and *K_low_* were sourced from RegioSQM, which comprises a collection of electrophilic aromatic substitution reactions for a total of 552 reactions (*38*). The negative examples are not provided in the original dataset, but they can be easily generated by repositioning the halogen atom in the product to an incorrect location, as illustrated in Fig. 1 (left). Between the two subsets of the data, *K_high_* contains all the positive reactions available in the dataset, whereas *K_low_* contains only 10% of them. Table 2 clarifies the number of samples in each split for the two subsets, which were generated following random sampling and a 40:30:30 ratio. The validation set from one randomly selected split was used for hyperparameter selection in the RL models, whereas the test set was used for final model comparisons.

### HiTEA dataset

The HiTEA dataset was prepared with standard preprocessing steps such as the removal of missing values, the canonicalization of smiles, and the compilation of the entire reaction through concatenation. The UV area yield was used to determine the observed reaction yield, which was labeled as positive for reactions with yield higher than one and negative for all the other reactions. The dataset was split in two subsets, replicating the *K_high_* and *K_low_* subsets for the experiments, with *K_high_* containing all the positive reactions available in the dataset and *K_low_* only a subset corresponding to 10% of them. Different random splits were created by random sampling with a 40:40:30 ratio. The performance was monitored during training on the validation set. The best hyperparameter configuration was chosen on this set and ultimately tested on the test set.

### Models

The baseline model for all experiments, called the forward model, is the forward reaction prediction model trained on the USPTO, which acts as the π_ref_ in the RL training (*45*). The model is a transformer model trained for 102,500 steps with PyTorch Lightning (PL) (*46*) to predict the outcome of the reactions (*47*–*49*). Predictions were generated using a beam search approach with 10 beams. The performances on the USPTO test set and the entire RegioSQM20 dataset are reported in Table 3. For instance, we assess the accuracy of the forward predictions by evaluating the top 1 and top 2 positive accuracy, namely, how often the correct result is included in the top 1 and top 2 predictions. In addition, we count the number of invalid molecules generated in both settings. The FT model is the forward model trained with fine-tuning on RegioSQM20 with Maximum Likelihood Estimation (MLE). Details on the equation are provided in the Supplementary Materials. The RL model is implemented to improve the performance of the forward model and is implemented in PL. Except for the HiTEA experiments, where additional tuning was needed to contrast domain shift, the RL model is directly applied to the forward model without any fine-tuning on the RegioSQM positives. The base reward model, called base model, is an ALBERT architecture pretrained using Huggingface. This model was then tuned with classification tuning, and SVM models were built with the scikit-learn toolkit on both the raw and classification tuned embeddings. The value network for the baseline model is a PyTorch MLP with two linear layers, separated by a ReLU activation function, and a sigmoid applied at the output.

**Table 3.** Base language model performance. Positive accuracy (pos. acc.) and % of predicted invalids.

Metric	USPTO		RegioSQM20	
	Top 1	Top 2	Top 1	Top 2
Pos. acc. ↑	59.43	67.78	52.90	69.20
% Invalids ↓	1.36	4.74	1.81	3.99

### Details on the methods

Additional details on the methods, including details on the adaptation of RLHF to the forward reaction prediction and the reward function, are given in the Supplementary Materials.

### Statistical analysis

Statistical validation of the results is performed by repeating the experiments on five different data splits and with different initialization seeds. Confidence intervals representing the SD are reported in the Results section.

## Supplementary Materials

### This PDF file includes:

Supplementary Materials and Methods Supplementary Results Figs. S1 to S8 Tables S1 and S2
