# Supplementary material for: Negative chemical data boosts language models in reaction outcome prediction
Source: Sci Adv. 2025 Jun 13;11(24):eadt5578. doi: 10.1126/sciadv.adt5578 (PMC12164950; doi:10.1126/sciadv.adt5578)
Supplement: Supplementary file 1 — Supplementary Materials and Methods Supplementary Results Figs. S1 to S8 Tables S1 and S2 [file sciadv.adt5578_sm.pdf]

Science Advances
AAAS

# Supplementary Materials for

## Negative chemical data boosts language models in reaction outcome prediction

Alessandra Toniato *et al.*

Corresponding author: Alessandra Toniato, alessandra.toniato94@gmail.com;
Mara Graziani, mara.graziani@ibm.com

*Sci. Adv.* **11**, eadt5578 (2025)
DOI: 10.1126/sciadv.adt5578

**This PDF file includes:**

Supplementary Materials and Methods
Supplementary Results
Figs. S1 to S8
Tables S1 and S2

## Additional details on the materials and methods

Adapting RLHF to learn from negative reactions

We adapted the RLHF framework to train forward prediction models using negative reaction data. The approach comprises three phases: 1. supervised fine-tuning (SFT), 2. learning of the reward model from preferences, and 3. reinforcement learning parameter optimization (RL).
In Phase 1, the objective of SFT is to develop an initial language model ($\pi_{SFT}$) that would serve as a stable foundation for subsequent RL training. This approach mitigates the inefficiencies associated with random weight initialization.
Phase 2 involves constructing a reward model capable of distinguishing between preferred and dis-preferred outcomes, that are, in our case, positive and negative reactions. Traditionally, this model is a transformer architecture trained on pairs positive and negative samples, predicting the log probability that a given outcome would be favoured by human evaluators (*42*).
In Phase 3, during the RL optimization, the model is refined by adjusting the rewards assigned to predicted sequences. These rewards, derived from the reward model, modulate the log probability of predictions, thereby encouraging or discouraging specific outcomes as defined by the learned preferences.
We detail below the specific adaptations made to this optimization framework to apply it effectively to chemical reaction data.

Supervised Fine-Tuning.

We fine-tune a vanilla transformer model to predict the outcomes of chemical reactions (*43*–*46*). An input sequence consists of the reactants and reagents ($x$), while the target is their corresponding product ($y$). Both reactants and products are encoded as SMILES strings (*47,48*). To enhance model robustness, the input molecules are augmented by rotating the starting atom in the SMILES notation during training. The model is pre-trained using the open-source collection of reactions from the USPTO dataset (*7*) and fine-tuned on RegioSQM (*6*). The training is conducted with teacher forcing, optimizing the model parameters according to the maximum likelihood estimation (MLE) objective:

$$L_{MLE}(y_{1:N}|x,\theta) = -\sum_{j=1}^{N}\left(\log \pi\left(y_j|y_{<j},x,\theta\right)\right) \qquad (1)$$

Where N is the length of the sequence and $\pi$ is the output of the transformer network, hence the predicted probability over a vocabulary of tokens. We refer to this model as the *forward model*, $\pi_{ref}$.

Reward Model Training.

The training of the reward model required significant adaptation from traditional RLHF approaches due to the unique challenge of learning from negative reactions in a low-data regime. Unlike standard RLHF scenarios where outcomes can be ranked based on varying levels of preference, here, predictions are strictly binary—either correct or incorrect. To handle this, we assign binary labels to each reaction, with 0 indicating an incorrect prediction

and 1 indicating a correct prediction. Given the limited size of our dataset, using a large transformer model to differentiate between correct and incorrect predictions would risk poor generalization. To overcome this, we first pre-train an ALBERT model architecture using Masked Language Modelling (MLM) on the USPTO dataset, obtaining the *base model* for the reward prediction. The base model's encoding is used to generate embeddings for RegioSQM, which serve as the foundation for the first modelling strategy. These embeddings are used to train support vector machines (SVMs) to distinguish between positive and negative reactions. In the second modelling strategy, we perform classification-tuning of the base model. We use the USPTO validation to generate three predictions per sample using the baseline forward model via beam search. The so obtained data are then curated into a classification dataset by combining all the incorrect predictions representing artificial negatives and the original positive samples.

Reinforcement Learning Optimization.

We optimized the language model using the REINFORCE Policy-Gradient method (*32*), incorporating several key modifications. Specifically, we introduced a regularization term into the loss function and estimated the value function baseline using a lookup table, as described in detail below. The optimization problem is formulated by combining two components: an RL term that computes the expected reward value and a regularization term based on Kullback-Leibler (KL) divergence, modulated by the parameter $\beta$:

$$\max_{\pi_\theta} \mathbb{E}_{x\sim X,\tilde{y}\sim\pi_\theta(\tilde{y}|x)}[\rho(x,\tilde{y})] - \beta D_{KL}[\pi_\theta(\tilde{y}|x)||\pi_{ref}(\tilde{y}|x)] \quad (2)$$

Here, $x$ represents the input dataset consisting of reactants and reagents, while $\tilde{y}$ denotes the product SMILES predicted by the model $\pi_\theta$ and $y$ corresponds to the ground truth target. The term $\pi_{\mathrm{ref}}$ refers to the reference model used for computing the KL divergence, which, in our case, is the forward model in Equation 1. The sequence reward is denoted by $\rho(x,y)$. This formulation leads to the following loss function:

$$L = \sum_j^N \Psi_j * \log \pi_\theta(\tilde{y}_j|x,\tilde{y}_{<j}) - \beta \log \frac{\pi_\theta(\tilde{y}_j|x,\tilde{y}_{<j})}{\pi_{ref}(\tilde{y}_j|x,\tilde{y}_{<j})} \quad (3)$$

The regularization term is designed to reduce the parameter deviation from the reference forward model obtained in Eq. 1, while preserving generation diversity as in (*36*). The $\beta$ parameter, which controls the strength of the regularization is dynamic.

Unlike the original REINFORCE algorithm, where $\Psi_j$ represents the step reward assigned to a specific action, we assign the reward to the tokens of the predicted product. Specifically, we apply the sequence reward $R$ to each individual token, i.e. $r(x,\tilde{y}_j) = R$. Moreover, to

stabilize training and the variance of the gradients, we incorporate the computation of a baseline value function, into the reward calculation. The expression for the step reward is given by:

$$\Psi_j = R - b(x, \tilde{y}_j) \qquad (4)$$

For the baseline model $b(x,\tilde{y}_j)$, we extract the final hidden outputs of the decoder during training and use them to train a Multi-Layer Perceptron (MLP) to predict the expected return value of the partial SMILES sequence.

The expected return, represented by the value function *V*, is given by:

$$b(x, \tilde{y}_t) = V(x, \tilde{y}_t) = \mathbb{E}_{\tilde{y}_{s_{t+1:\infty}}}[\sum_{0}^{\infty} r_{t+l}] \qquad (5)$$

The conventional formulation of the value function, as described in Equation 5, relies on step rewards that are not applicable in our context. To address this, we implement augmentation strategies for SMILES within the training dataset and estimate the expected return accordingly. For instance, for each training sample, we generate partial sequences comprising the input and progressively larger portions of the target. We then assign a probability to each sequence based on the frequency with which those partial strings lead to either a positive or negative reaction outcome. This approach assumes equal likelihood for each positive reaction and its corresponding negative instances. The resulting probabilities are stored in a lookup table, which is subsequently used as the target for training the value network.

Figure S8 illustrates the construction of the lookup table through examples. Starting from the top left corner, we construct the prior lookup table used to compute the value function prior. Each reaction in the training set (both positive and negative) is augmented by randomizing the product using the RDKit library (*48*) (product augmentation). This augmentation is performed three times per product, as additional augmentations did not result in significant improvements in model performance. After augmentation, each product is converted into partially completed strings (partial strings enumeration). The final lookup table is constructed by collecting the counts and rewards associated with all partial strings.
The lookup table is updated during training to keep track of the information associated to the new predicted positives. The two tested strategies are illustrated on the right-hand side of Figure S1. In the cumulative strategy, after a lookup update cycle that lasts for a fixed number of epochs, the predictions for the entire training set are collected and added to the table in terms of count and score. In the replacement strategy, after each iteration cycle, the predictions are used to replace the prior scores and counts for the specific partial strings. The frequency of updates to the lookup table, and thus the length of the lookup update cycle, is a parameter we tune during training. A comparison of the results obtained by implementing each of these strategies is presented in Figure S4.

Finally, the value network is trained to minimize the following non-linear regression objective:

$$\min_{\xi} \sum_{n=1}^{N} ||V_{\xi}(x, \tilde{y}_t) - \tilde{V}_n||^2 \quad (6)$$

Here, *n* indexes all steps in a batch of trajectories, encompassing all tokens in all predicted products of a batch. In many approaches, $V_{\tilde{n}}$ coincides with either the step reward or the sequence reward. In this work, however, $V_{\tilde{n}}$ is defined as the frequency computed by referencing the count and score from the lookup table for the corresponding partially completed SMILES. After computing Equation 4, all rewards are whitened before being multiplied by the log probability.

Details on reward model training

Here we report the details on the training of the reward model described in the Methods.

For the $K_{high}$ scenario we realized that all the training positive reactions were already observed at the beginning of training. In this case, the table was efficiently implemented as a hash table that includes all the partial sequences leading to a positive. This is feasible due to the nature of the forward prediction task since each input has only one possible canonical output. The 100 cumulative step strategy was used to update the table during training.

For the $K_{low}$ dataset, the base reward model was classification tuned to maximize the separability of positive and negative reactions. The configuration files used for the training are available in the code repository.

Baseline term in the value function

Different value functions were evaluated in this study, of which some implemented a baseline term. Figure S4 compares the top 1 accuracy obtained by implementing different baseline functions, as evaluated on the $K_{high}$ test dataset. The results are averaged across five random splits, with standard errors reported in the colored shading.

Among the analyzed strategies there are: (i) a baseline function that returns random values, RANDbaseline; (ii) no baseline function at all, NObaseline; (iii) a cumulative baseline and (iv) a replacement baseline. The cumulative (iii) and replacement (iv) baseline strategies are described in the Methods section of the main paper. The no baseline strategy (ii) uses only sequence rewards as partial rewards ($\Psi_j = R$), and the random baseline (i) assigns a random value between 0 and 1 to $b(x,\tilde{y}_j)$ (Equation 4) of each token. In this analysis, the lookup table updates were performed every 100 epochs.

Hyperparameters grid search list

We conducted a comprehensive hyperparameter search to optimize model performance across different settings. For the SVM model, we explored various values for the regularization parameter C, including values between $1e-3$ and 100. We also evaluated different kernel types, specifically

radial basis function (RBF), polynomial, and sigmoid kernels. Additionally, we tested multiple settings for the gamma parameter. For the base model, we tuned key hyperparameters such as learning rate, batch size, weight decay, and the choice of optimizer to ensure stable and efficient training. Similarly, for the RL-tuned model, we adjusted the learning rate, batch size, and baseline batch size while experimenting with different baseline types to optimize policy learning. We also fine-tuned the KL reference and beta parameters to balance exploration and adherence to the reference policy.

## Supplementary results

<u>FT against RL in high positive data regime</u>

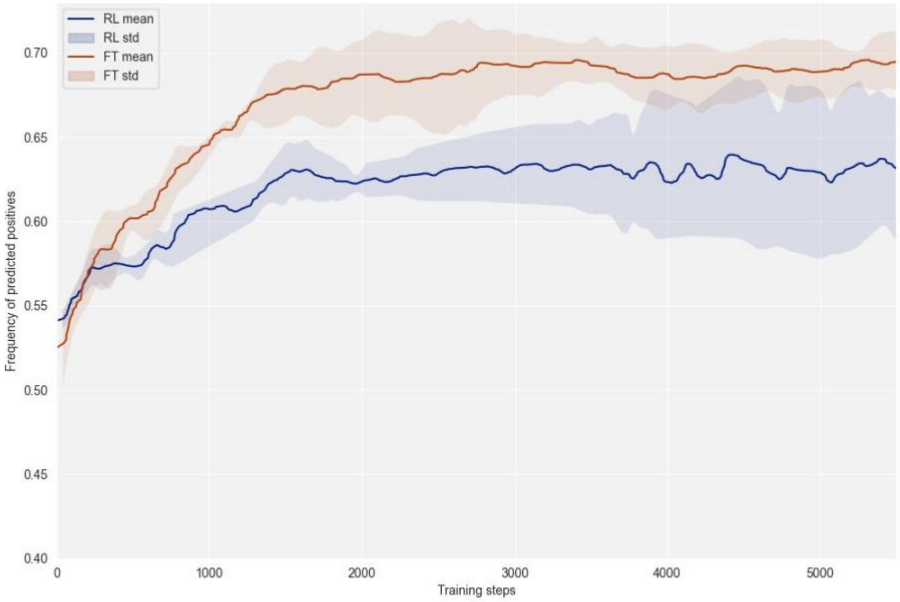


**Figure S1**: **FT against RL on $K_{high}$.** Performance of FT and RL on $K_{high}$, from which we derived the upper bound of FT on $K_{high}$ as a dotted magenta line. The ratio of positive and negative reactions here is high (0.3 against 0.03 of $K_{low}$), and the RL model is considerably less effective than FT. This is expected, as the RL depends solely on its own predictions to identify the positives, rather than directly observing the ground truth for each positive instance as in FT. The learning curves in this figure are obtained by implementing the cumulative value function with an update rate of every 100 epochs for $K_{high}$ and every 50 epochs for $K_{low}$. The update frequency was tuned as a hyperparameter in both cases.

<u>FT performance variability</u>

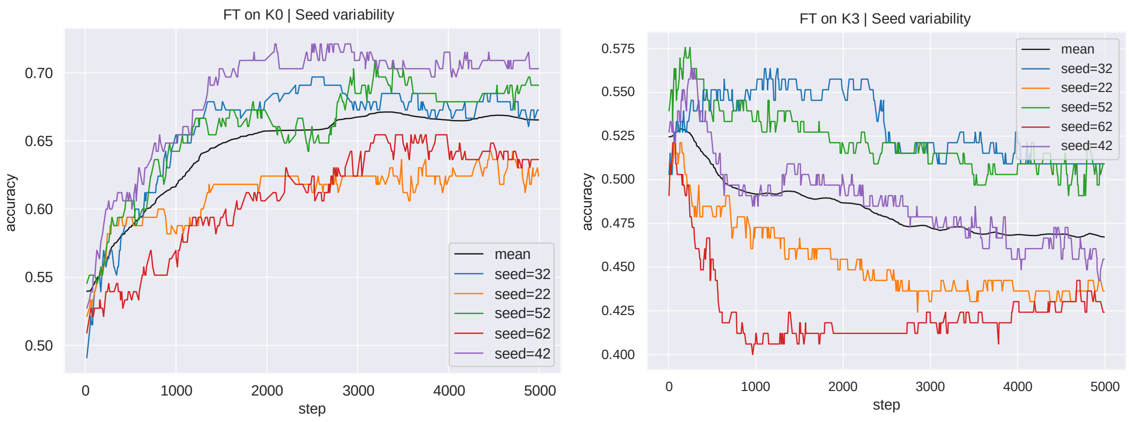


**Figure S2**: **Positive accuracy of FT**. Results from training on data splits obtained from five starting seeds for $K_{high}$ (left) and $K_{low}$ (right). Performance variability is observed in the FT baselines for both K high and K low. No clear difference appears in the consistency of reporting negatives in the test data used for the splits corresponding to high and low performance, seed 52 and seed 62, respectively. The dataset is constructed by associating a different number of negative examples to each positive, with the range of available negatives going from zero to eight. Similarly, there are negatives with no associated positives in both data splits.

Impact of classification tuning to the SVM in the reward model

**Table S1**: Train and Validation positive accuracy of the SVM models used for the reward function.

| **Positive Accuracy** | $\mathbf{K}_{high}$ | $\mathbf{K}_{low}$ |
|---|---|---|
| Train | 0.94 ± 0.05 | 0.87 ± 0.1 |
| Validation | 0.76 ± 0,07 | 0.65 ± 0.04 |

**Table S2**: Average Euclidean distance between point pairs taken from RegioSQM and USPTO. The standard deviation is reported in brackets. We report the positive-to-positive (positive → positive) and the positive-to-negative (positive → negative) distances as calculated in the space spanned by the first 72 principal components of the PCA, which account for 95% of the explained variance. The tuned positive-to-negative is higher of 1.54 points than the positive-to-positive distance, showing that classification-tuning enhances the separability between the two classes.

| USPTO to RegioSQM sample pairs | base model mean (std) | classification-tuned model mean (std) |
|---|---|---|
| positive → positive | 23.56 (±3.27e-4) | 18.52 (±1.67e-3) |
| positive → negative | 23.43 (±3.33e-4) | 20.06 (±1.26e-3) |
| absolute difference | 0.13 | **-1.54** |

Reward modelling approaches

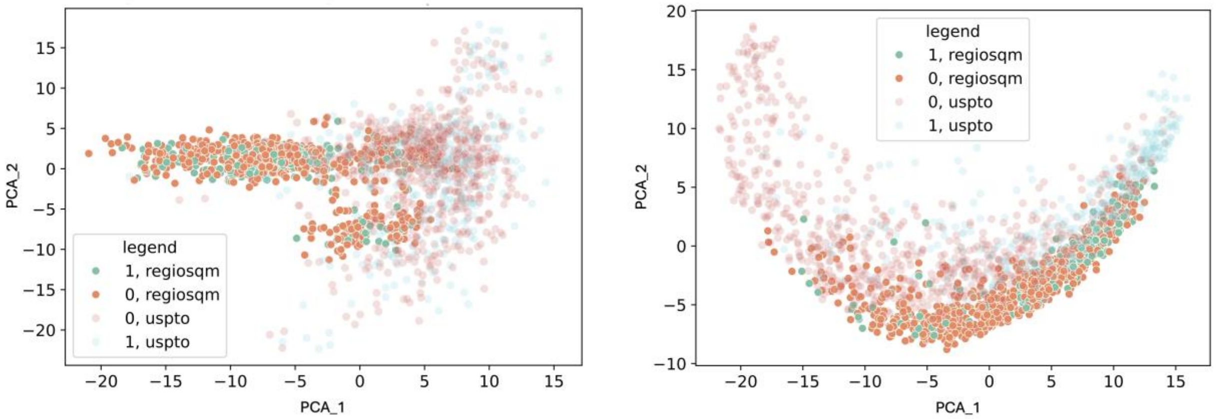


**Figure S3**: Projection on the two principal components of the positive and negative samples from RegioSQM and USPTO. On the left, the samples are obtained from the base model representation space. Positives and negatives are scattered without a precise structure, and the two datasets seem to belong to separate clusters. On the right, the samples are in the representation space obtained by classi fication-tuning. Positive reactions are organized along the first component of the variance, clustering on the right handside of the figure. This condition is ideal for classification, favoring the learning of the SVM.

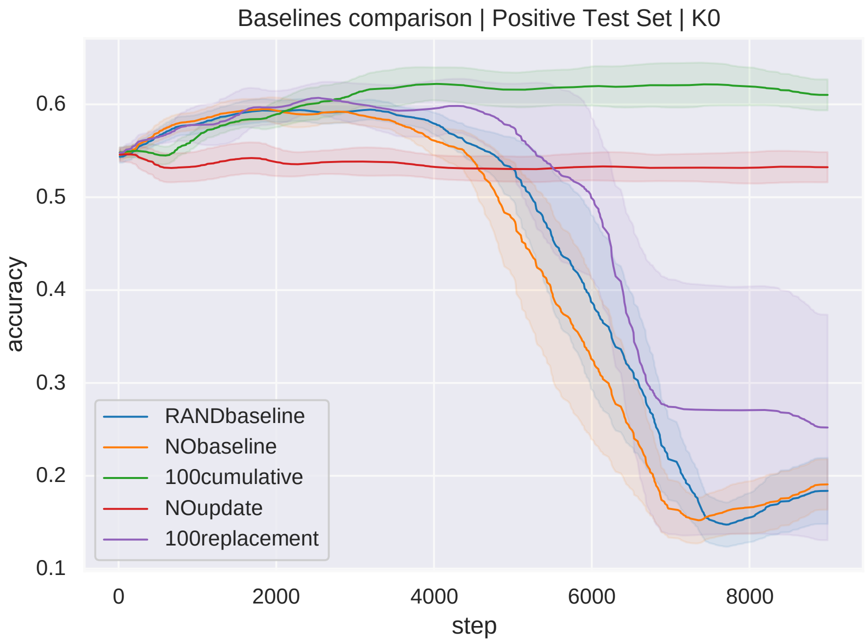


**Figure S4**: Comparison of different baseline approaches for the RL experiment.

<u>Applicability to High Throughput Experimentation Data</u>

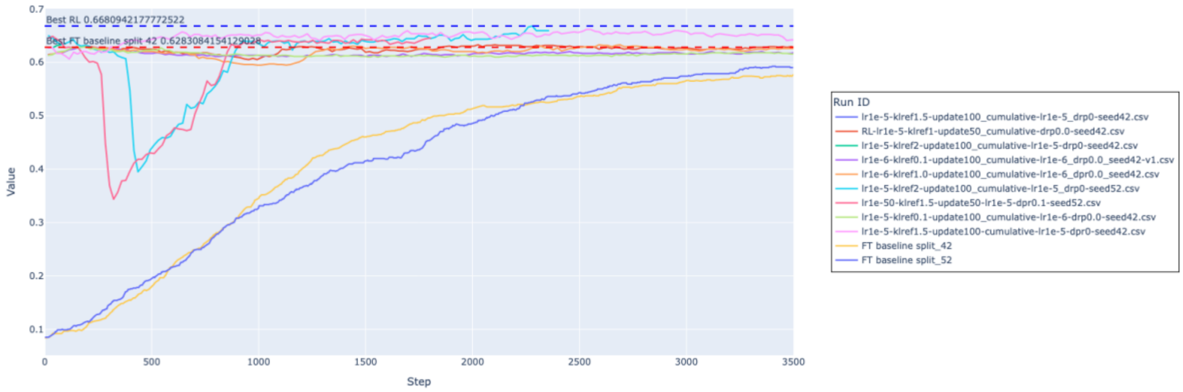


**Figure S5**: Performance curves on the validation set for training both the RL and FT models across different random seeds and hyperparameter configurations of the RL model. The RL pipeline was applied to the best-performing FT model checkpoint to further enhance performance, which explains why the RL curves start at 60% validation accuracy. A consistent improvement in performance is observed across various hyperparameter settings and seeds.

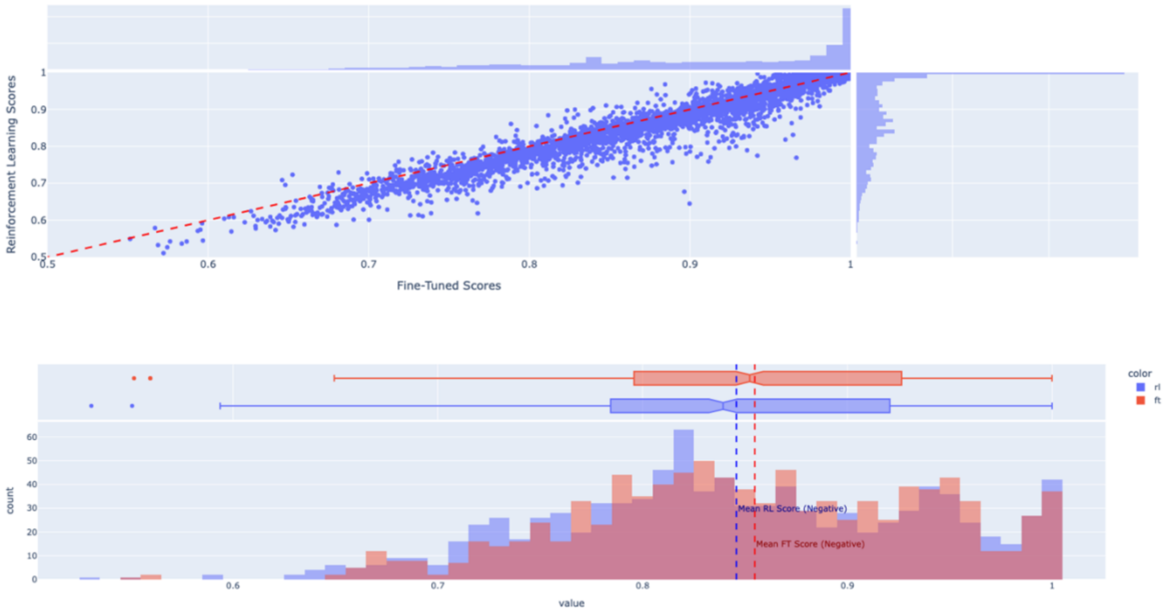


**Figure S6**: Comparison of confidence scores in the FT and RL model for the top 10 predictions. Top: relative deviation of confidence scores obtained by the FT and the RL model on the test set. Bottom: Distribution of the confidence scores for the predictions of low yield (i.e. negative) reactions. The RL model presents, on average, lower confidence scores than the FT counterpart.

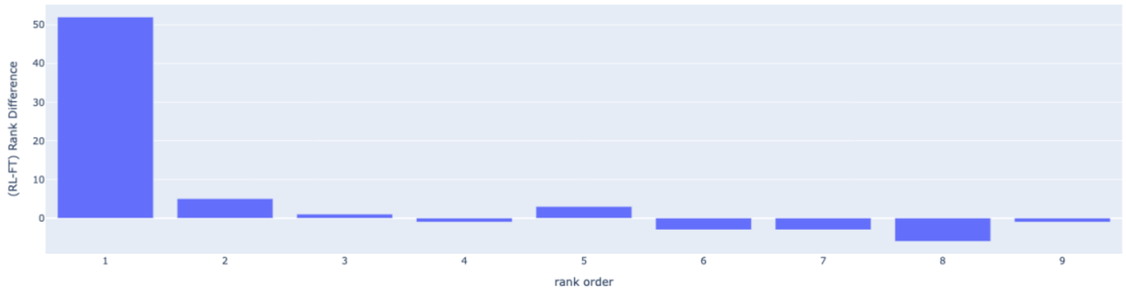


**Figure S7**: Impact of the RL to the ranking of the top 10 test predictions for high yield (i.e. > 1) reactions, expressed as the ranking difference observed in the RL model as opposed to the FT model. The FT model tends to rank positive reactions lower than the RL model, which, in contrast, ranks more positive predictions higher. This shift results from the exploration-exploitation trade-off inherent in the RL pipeline and depends on the reward model's ability to identify potentially high-yield reactions as predicted by the model.

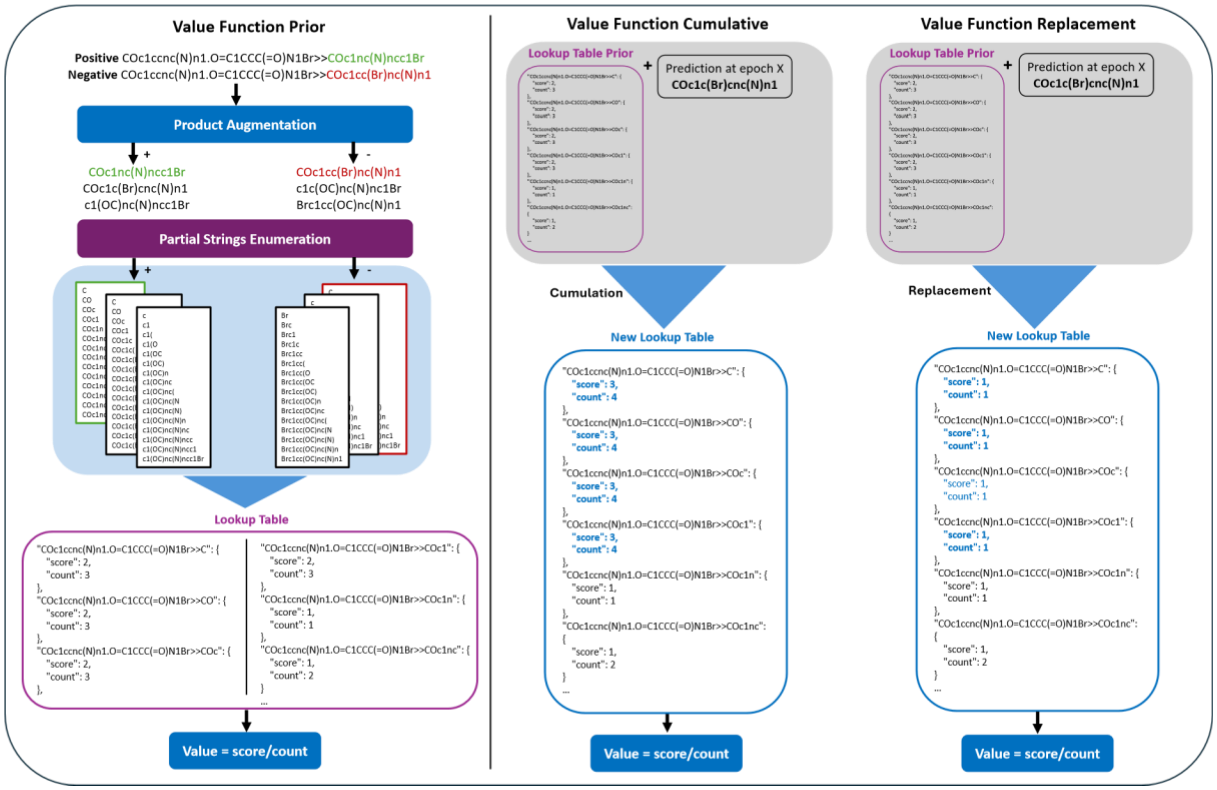


**Figure S8**: **Lookup table construction.** On top the Value Function prior lookup table used at the beginning of training. On the bottom the two different strategies for the update of the table during training (cumulative and replacement).
